# Insulin resistance, vitamin D status, and cardiovascular risk in a general population cohort

**DOI:** 10.3389/fnut.2026.1868349

**Published:** 2026-07-17

**Authors:** Sisi Luan, Luning Chen, Juanjuan Zhu, Bi Tang, Xuelian Zhang, Wenke Cheng

**Affiliations:** 1 Department of Endocrinology, Beijing Tongren Hospital, Capital Medical University, Beijing, China; 2 Department of Pharmacy, Lintong Rehabilitation and Recovery Center, Xi'an, China; 3 Beijing Diabetes Institute, Beijing Tongren Hospital, Capital Medical University, Beijing, China; 4 Department of Cardiology, The First Affiliated Hospital of Bengbu Medical University, Bengbu, China; 5 School of Life Science, Anhui Provincial Key Laboratory of Tumor Evolution and Intelligent Diagnosis and Treatment, Anhui Province Key Laboratory of Immunology in Chronic Diseases, Bengbu Medical University, Bengbu, China

**Keywords:** insulin resistance, major adverse cardiovascular events, triglyceride-glucose index, UK biobank, vitamin D

## Abstract

**Aims:**

Insulin resistance and vitamin D deficiency have each been implicated in cardiovascular disease development, yet their joint effects on major adverse cardiovascular events (MACE) remain unclear. We aimed to investigate the independent and combined associations of the triglyceride–glucose (TyG) index and serum 25-hydroxyvitamin D [25(OH)D] levels with the risk of MACE.

**Methods:**

This retrospective analysis was conducted using data from the UK Biobank, a large prospective cohort comprising 335,206 participants who were free of cardiovascular disease at baseline. The TyG index and serum 25(OH)D concentrations were assessed at enrollment. MACE was defined as a composite of ischemic heart disease, stroke, sudden cardiac arrest, and cardiovascular mortality. Multivariable Cox proportional hazards models were applied to estimate hazard ratios (HRs) and 95% confidence intervals (CIs). Additive and multiplicative interactions were evaluated using the relative excess risk due to interaction (RERI), attributable proportion (AP), and synergy index (SI). Population-attributable cases and excess incidence rates were also estimated.

**Results:**

During a median follow-up of 13.77 years, 33,532 incident MACE events occurred. After multivariable adjustment, both a higher TyG index and lower serum 25(OH)D levels were independently associated with an increased risk of MACE. Participants with concomitant high TyG (≥9.4) and vitamin D deficiency (<50 nmol/L) exhibited the highest risk (HR 1.26, 95% CI: 1.18–1.35). A significant additive interaction was observed (RERI 0.074, 95% CI 0.017–0.131; AP 0.059, 95% CI 0.014–0.104; SI 1.39, 95% CI 1.01–1.78). This joint exposure accounted for 4,369 attributable MACE cases and an excess incidence rate of 35.4 per 10,000 person-years.

**Conclusion:**

Both an elevated TyG index and vitamin D deficiency are independently associated with an increased risk of MACE. Moreover, a significant statistical interaction between these factors further exacerbates cardiovascular risk in the general population. These findings identify the concomitance of insulin resistance and vitamin D deficiency as a high-risk phenotype, warranting further research to elucidate its clinical relevance.

## Lay summary

Vitamin D deficiency and insulin resistance are both linked to increased cardiovascular risk. The triglyceride-glucose (TyG) index is a validated surrogate marker of insulin resistance and an emerging predictor of major adverse cardiovascular events (MACE). However, their combined impact and potential joint effects on cardiovascular outcomes in the general population have not been fully explored.

- Interactive risk: a significant statistical interaction was observed between elevated TyG index (≥9.4) and vitamin D deficiency (<50 nmol/L), increasing MACE risk beyond their individual effects.
- Population burden: dual exposure accounted for over 4,369 attributable MACE cases and an excess incidence rate of 35.4 per 10,000 person-years.
- Subgroup effect: the joint effect was more pronounced in participants without hypertension, diabetes, or obesity, highlighting risk in otherwise metabolically healthy individuals.
- Clinical implication: the combination of elevated TyG index and vitamin D deficiency identifies a subgroup at amplified cardiovascular risk.

## Introduction

Cardiovascular disease (CVD) remains the leading cause of morbidity and mortality worldwide, accounting for a substantial proportion of premature deaths despite advances in prevention and treatment strategies. Metabolic dysregulation and chronic low-grade inflammation play central roles in the initiation and progression of atherosclerosis and related cardiovascular complications. Identifying accessible biomarkers that capture these pathophysiological processes and improve risk stratification remains a major public health priority.

Insulin resistance represents a core metabolic abnormality underlying cardiometabolic disorders and has been consistently linked to adverse cardiovascular outcomes (1, 2). The triglyceride–glucose (TyG) index, derived from triglyceride and glucose concentrations, has emerged as a simple and reliable surrogate marker of insulin resistance (3–5). Accumulating evidence indicates that a higher TyG index is associated with an increased risk of major adverse cardiovascular events (MACE) in both general and high-risk populations (6). Compared with traditional indices such as the homeostasis model assessment of insulin resistance (HOMA-IR), the TyG index offers advantages in availability, reproducibility, and predictive performance, making it particularly suitable for large-scale population studies (7). Despite its established predictive value, the interactions between the TyG index and other metabolic factors, as well as the underlying mechanisms, remain complex and warrant further investigation (8, 9).

Beyond its classical functions in calcium and phosphorus metabolism, vitamin D influences multiple biological processes relevant to cardiovascular regulation, including endothelial function, lipid metabolism, inflammatory signaling, and the renin–angiotensin system (10). Epidemiological studies have linked vitamin D deficiency to an increased risk of atherosclerosis, hypertension, diabetes, and adverse cardiovascular outcomes (11). Mechanistically, vitamin D deficiency may promote vascular dysfunction through impaired nitric oxide bioavailability, enhanced inflammatory responses, and dysregulation of metabolic pathways (12). Notably, vitamin D deficiency and insulin resistance frequently coexist, particularly among individuals with obesity and metabolic abnormalities, suggesting a potential biological interplay between these two conditions (13). Experimental and clinical evidence indicates that vitamin D may influence insulin secretion and sensitivity through interactions with the vitamin D receptor and downstream metabolic signaling pathways (14, 15). These findings support a plausible mechanistic link between vitamin D status and insulin resistance.

Despite growing evidence supporting the independent associations of both the TyG index and vitamin D deficiency with cardiovascular risk, their combined effects on major adverse cardiovascular events have not been well characterized. In particular, it remains unclear whether vitamin D deficiency modifies or amplifies the cardiovascular risk associated with insulin resistance, and to what extent their coexistence contributes to the overall population burden of cardiovascular disease. Therefore, this study leveraged the UK Biobank cohort to explore the combined influence of the TyG index and vitamin D deficiency on MACE and assess their joint effects.

## Method

### Study design and population

This study utilized data from the UK Biobank, a large, prospective cohort designed to examine the determinants of major chronic diseases in adults. Between 2006 and 2010, 502,357 individuals aged 40–69 years were recruited from across the United Kingdom. The detailed study design and methodology are available elsewhere (16). At baseline, participants completed self-administered touchscreen questionnaires covering sociodemographic characteristics, lifestyle behaviors, and medical history, followed by standardized physical examinations and venous blood sampling. Long-term follow-up was achieved through linkage to national health-related datasets, including hospital inpatient records, cancer registries, and death certificates. The UK Biobank received ethical approval from the North West Multi-Center Research Ethics Committee (reference 11/NW/0382), with all participants providing written informed consent.

Individuals with cardiovascular disease at baseline, including ischaemic heart disease (IHD), stroke, heart failure, valvular disease, cardiomyopathy, arrhythmias, or cardiac arrest, as well as those with cancer or who were pregnant (*n* = 90,779), were excluded (Supplementary Figure S1). Pregnancy and cancer were excluded due to their frequent association with vitamin D deficiency and acute metabolic disturbances, which may confound associations between vitamin D, the TyG index, and cardiovascular outcomes (17, 18). Additional exclusions were made for participants with missing data on the TyG index (*n* = 59,406), vitamin D levels (*n* = 16,964), or enrolment date (*n* = 2). After exclusions, 335,206 participants remained for the primary analysis. These participants were categorized into four exposure groups based on binary classifications of TyG index and vitamin D status: low TyG and high vitamin D (*n* = 88,677); low TyG and low vitamin D (*n* = 91,998); high TyG and high vitamin D (*n* = 59,950); and high TyG and low vitamin D (*n* = 94,581).

### Ascertainment of vitamin D status

Vitamin D status was assessed by measuring serum 25-hydroxyvitamin D [25(OH)D], the primary circulating biomarker of vitamin D. Serum 25(OH)D concentrations were determined from baseline blood samples using a chemiluminescence immunoassay (LIAISON XL, DiaSorin Ltd., UK), with an analytical range of 10–375 nmol/L. All measurements were performed by the UK Biobank laboratory, following standardized quality control procedures. The intra-assay coefficients of variation ranged from 5.0 to 6.1%, with results externally validated against reference standards, demonstrating full concordance. Detailed assay protocols have been described previously (19).

In line with the Endocrine Society Clinical Practice Guidelines and given the low prevalence of serum 25(OH)D concentrations ≥75 nmol/L in this cohort, vitamin D status was categorized for analytical purposes as deficient (<50 nmol/L) or sufficient (≥50 nmol/L) (20).

### Assessment of TyG index

Random peripheral venous blood samples were collected at baseline in accordance with the UK Biobank protocol. Serum triglyceride and glucose concentrations were measured using enzymatic methods on the Beckman Coulter AU5800 platform. The average coefficients of variation for triglycerides and glucose were <3 and <2%, respectively, ensuring high analytical precision. The TyG index was calculated using the following formula: TyG = ln [triglycerides (mg/dL) × glucose (mg/dL) / 2] (21).

### Definition and ascertainment of MACE

The incidence of cardiovascular events in our study was ascertained using the UK Biobank First Occurrences dataset (Category 1,712), which integrates information from primary care, hospital inpatient, death registry, and self-reported data, all mapped to 3-character International Classification of Diseases, Tenth Revision (ICD-10) codes. The primary outcome of this study was MACE, defined as a composite of nonfatal IHD (I20–I25), nonfatal stroke (I60–I64), sudden cardiac arrest (I46), and cardiovascular mortality (I00–I99). Secondary outcomes included the individual components of MACE—specifically IHD, stroke, and sudden cardiac arrest—as well as cardiovascular mortality.

Incident events were identified through linkage with hospital inpatient records from the Hospital Episode Statistics for England, the Scottish Morbidity Record, and the Patient Episode Database for Wales. Sudden cardiac arrest was also captured using the UK Biobank's “first occurrence” algorithm, which integrates hospital admission and mortality data. Cardiovascular mortality, defined as death attributable to any cardiovascular cause, was determined through linkage with national death registries, including NHS Digital (for England and Wales) and the NHS Central Register (for Scotland). Dates and causes of death were obtained from official death certificates. Participants were followed from the date of enrollment until the occurrence of MACE, death, loss to follow-up, or administrative censoring on December 19, 2022, whichever occurred first.

### Assessment of covariates

Baseline covariates were collected using standardized touchscreen questionnaires, physical measurements, and biomarker assays. Demographic variables included age, sex, and self-reported ethnicity (White vs. non-White). Socioeconomic status was assessed using education level (none, high school or below, college or above, other professional qualifications, or missing), average total household income (<£51,999, ≥ £52,000, or missing), and the Townsend Deprivation Index (TDI), a validated composite measure based on neighborhood-level unemployment, car ownership, home ownership, and overcrowding, with higher values indicating greater deprivation.

Lifestyle factors included smoking status (never, ever, or missing), alcohol consumption (never, ever, or missing), dietary quality score, and physical activity. A cumulative dietary risk score was calculated to assess diet-related risk factors, based on a previously established approach in the UK Biobank. Nine food items—processed meat, red meat, total fish, milk, spreads, cereal, table salt, water, and fruit and vegetables—were classified as either meeting or failing to meet UK/European dietary recommendations. Each unfavorable dietary component was assigned a score of 1 point, resulting in a total score ranging from 0 (healthiest) to 9 (unhealthiest) (22). Total physical activity was estimated in metabolic equivalent (MET) minutes per week, based on a modified version of the International Physical Activity Questionnaire (23). Hypertension and diabetes mellitus (DM) were defined using self-reported physician diagnoses and linked hospital data, with antihypertensive medication use also considered indicative of hypertension. Medication use for lipid-lowering agents and insulin therapy was recorded. Frailty status was evaluated using a UK Biobank-adapted Fried phenotype model, incorporating weight loss, exhaustion, grip strength, walking speed, and physical activity. Participants were classified as non-frail, pre-frail, or frail based on criteria previously established in UK Biobank studies (24, 25).

Biomarkers, including triglycerides, glucose, glycated hemoglobin (HbA1c), total cholesterol, low-density lipoprotein cholesterol (LDL-C), and high-density lipoprotein cholesterol (HDL-C), were measured from random peripheral venous blood samples using enzymatic assays on the Beckman Coulter AU5800 analyzer. Estimated glomerular filtration rate (eGFR) was calculated using the 2021 CKD-EPI creatinine equation based on age, sex, and serum creatinine (26). Systolic (SBP) and diastolic blood pressure (DBP) were measured using an automated device, with two readings taken a few minutes apart. If automated readings were not available, a manual sphygmomanometer was used. BMI was calculated from measured height and weight. Missing data for continuous variables were imputed using the mean, while categorical variables with missing responses (e.g., “prefer not to answer”) were categorized separately to ensure analytical completeness.

A directed acyclic graph (DAG; Supplementary Figure S1) was constructed using DAGitty (www.dagitty.net) to identify potential confounders in the relationship between TyG/vitamin D and MACE risk (27). Based on this causal framework, the following variables were identified as minimally sufficient for adjustment: age, sex, race, BMI, physical activity, diet score, HbA1c, hypertension, diabetes, insulin use, lipid-lowering medication, smoking status, and alcohol consumption. Socioeconomic indicators (TDI, educational attainment, and average household income) were considered upstream determinants of TyG and vitamin D, primarily influencing through lifestyle mediators such as diet and physical activity. Following causal inference principles, these variables were excluded from the adjustment model to prevent overcontrol. Similarly, lipid profiles and blood pressure metrics (including total cholesterol, LDL-C, HDL-C, SBP, and DBP) were treated as downstream mediators and were also excluded from the adjustment set to avoid attenuation of the total effect estimate.

The covariates identified as minimally sufficient for adjustment were empirically evaluated using two criteria: (1) altering the exposure–outcome β coefficient by ≥ 10%; or (2) showing a statistically significant association with the outcome (*p* < 0.1) (28, 29). All selected covariates met both criteria and were retained in the final multivariable models.

### Statistical analysis

Participants were stratified based on TyG index and 25(OH)D levels. A binary cutoff of TyG at 9.4 was selected using the Youden index from receiver operating characteristic curve analysis, optimized for predicting MACE occurrence. Vitamin D status was categorized using a clinically relevant threshold of 50 nmol/L, aligning with established definitions of vitamin D deficiency. Participants were grouped according to these thresholds for subsequent comparative and survival analyses. Baseline characteristics were summarized across TyG and vitamin D categories. Continuous variables were expressed as mean ± standard deviation (SD) and compared using the Student's t-test. Categorical variables were presented as frequencies (percentages) and compared using the Chi-square test.

Kaplan–Meier curves were generated to visualize cumulative incidence across TyG and vitamin D categories, and log-rank tests assessed between-group differences. Time-to-event outcomes were analyzed using Cox proportional hazards models. TyG index and 25(OH)D levels were evaluated as both continuous (per unit and per SD increase) and categorical variables. Hazard ratios (HRs) and 95% confidence intervals (CIs) were estimated in multivariable models adjusted for age, sex, race, BMI, physical activity, HbA1c, diet score, smoking and alcohol status, hypertension, diabetes, insulin use, and lipid-lowering therapy. Proportional hazards assumptions were tested using Schoenfeld residuals and confirmed to be met.

Joint exposure analysis combined TyG index and vitamin D categories into a four-level variable. Additive interaction was assessed using relative excess risk due to interaction (RERI), attributable proportion (AP), and synergy index (SI), with 95% CIs calculated *via* the delta method. Multiplicative interaction was evaluated by including product terms in Cox proportional hazards models, with results expressed as hazard ratios (HRs) to quantify the strength of interaction between the four groups. A positive additive interaction was considered present when RERI > 0, AP > 0, or *S* > 1, with corresponding 95% CIs excluding null values. Multiplicative interaction was deemed significant if the interaction term yielded a *P*-value <0.05. Robust evidence of interaction was considered when both additive and multiplicative criteria were met.

Absolute risk metrics, including incidence rate ratios (IRRs), excess incidence rates (EIRs), attributable cases, and number needed to screen (NNS), were estimated using stratified Poisson regression models with robust standard errors. The log of person-years (PY) was included as an offset term, and stratification was performed by single-year age and sex combinations (age × sex strata) to adjust for potential confounding by age and sex. IRRs were calculated by comparing each joint exposure group (TyG and vitamin D) to the reference group (TyG <9.4 and 25(OH)D ≥ 50 nmol/L). The excess number of events attributable to the joint exposure was computed using the formula: Attributable cases = (1 – 1/IRR) × observed number of events. EIR was then derived by dividing the number of attributable cases by the total PY of follow-up in the corresponding exposure group, expressed per 10,000 PY. For clinical interpretation, NNS to prevent one MACE event was calculated as the inverse of EIR and expressed in PY.

To assess dose–response relationships, restricted cubic spline (RCS) regression was employed to model the associations between TyG or 25(OH)D levels and MACE risk. RCS with four knots were fitted at the 5th, 35th, 65th, and 95th percentiles of the exposure distribution, as recommended by Harrell FE (30). Non-linearity was evaluated using likelihood ratio tests by comparing the full spline model with a nested linear model. A two-sided *P*-value <0.05 was considered indicative of a statistically significant non-linear association.

Subgroup analyses were conducted by age, sex, race, BMI, physical activity, dietary risk score, hypertension, and diabetes status to assess potential effect modification. Sensitivity analyses were performed in the following ways: (1) excluding participants who developed MACE within the first 2 years of follow-up; (2) excluding participants with missing baseline covariates to evaluate robustness in a complete-case population; and (3) implementing multiple imputation *via* chained equations to address missing data, with results across imputed datasets pooled using Rubin's rules. and (4) fitting a series of progressively adjusted Cox models, sequentially incorporating season of assessment, fasting time, eGFR, and frailty level into the main model. This analysis aimed to determine whether the associations remained stable after accounting for seasonal variation, fasting status, renal function, and frailty status. All statistical analyses were conducted using R software (version 4.2.3). A two-sided *P*-value <0.05 was considered statistically significant.

## Results

Baseline characteristics of the participants are summarized in Table 1. The mean age of the study population was 55.7 ± 8.1 years, with 46.2% of participants being male. Compared to individuals with 25(OH)D ≥ 50 nmol/L, those with 25(OH)D <50 nmol/L were younger, had higher BMI, and displayed less favorable metabolic profiles, including elevated BMI, HbA1c, triglycerides, blood pressure, total cholesterol, and LDL-C, as well as lower physical activity levels and HDL-C (all *P* < 0.001). Similarly, participants with TyG ≥ 9.4 were more likely to be older, male, and white, and had higher BMI, blood pressure, and HbA1c, but lower physical activity levels and diet scores compared to those with TyG <9.4. Notably, individuals with a higher TyG index exhibited significantly greater prevalence of hypertension, diabetes, insulin use, and lipid-lowering therapy, along with marked differences in smoking and alcohol consumption patterns (*P* < 0.001 for all).

**Table 1:** Baseline characteristics of the study population stratified by the TyG index and 25(OH)D concentration.

Variables	Total	25(OH)D (nmol/L)			TyG		
		<50	≥50	*P*-value	<9.4	≥9.4	*P*-value
Sample size	335,206	186,579	148,627		180,675	154,531	
Age	55.7 ± 8.1	55. ± 8.1	56.5 ± 8	<0.001	54.8 ± 8.2	56.7 ± 7.9	<0.001
Men (%)	155,010 (46.24%)	87,157 (46.71%)	67,853 (45.65%)	<0.001	67,317 (37.26%)	87,693 (56.75%)	<0.001
White (%)	315,147 (94.02%)	169,765 (90.99%)	145,382 (97.82%)	<0.001	168,974 (93.52%)	146,173 (94.59%)	<0.001
TDI	−1.34 ± 3.06	−1.01 ± 3.21	−1.76 ± 2.81	<0.001	−1.38 ± 3.05	−1.29 ± 3.08	<0.001
BMI(kg/m^2^)	27.29 ± 4.73	27.89 ± 5.07	26.54 ± 4.13	<0.001	26.00 ± 4.34	28.81 ± 4.72	<0.001
Mean DBP	82.42 ± 10.07	83.01 ± 10.20	81.67 ± 9.85	<0.001	80.82 ± 9.99	84.28 ± 9.83	<0.001
Mean SBP	137.47 ± 18.44	137.85 ± 18.50	136.98 ± 18.35	<0.001	134.48 ± 18.47	140.96 ± 17.77	<0.001
Physical activity (MET-min/week)	2,669.32 ± 2,355.42	2,471.18 ± 2,224.82	2,918.06 ± 2,487.53	<0.001	2,749.47 ± 2,372.19	2,575.62 ± 2,332.18	<0.001
TC(mmol/L)	5.65 ± 0.98	5.69 ± 0.99	5.60 ± 0.97	<0.001	5.50 ± 0.94	5.83 ± 1.00	<0.001
HbA1c (mmol/mol)	35.75 ± 6.19	36.08 ± 6.86	35.34 ± 5.21	<0.001	34.59 ± 4.24	37.10 ± 7.67	<0.001
HDL-C(mmol/L)	1.45 ± 0.38	1.42 ± 0.37	1.48 ± 0.38	<0.001	1.59 ± 0.38	1.29 ± 0.30	<0.001
LDL-C(mmol/L)	3.53 ± 0.76	3.57 ± 0.76	3.48 ± 0.75	<0.001	3.38 ± 0.72	3.70 ± 0.77	<0.001
Diet score	5.07 ± 1.52	5.18 ± 1.52	4.93 ± 1.51	<0.001	4.95 ± 1.52	5.21 ± 1.50	<0.001
Fasting hours	3.8 ± 2.4	3.9 ± 2.6	3.7 ± 2.3	<0.001	3.9 ± 2.6	3.7 ± 2.3	<0.001
eGFR (mL/min/1.73 m^2^)	95.4 ± 12.8	96.2 ± 13	94.3 ± 12.6	<0.001	96.3 ± 12.3	94.4 ± 13.3	<0.001
Hypertension (*n*, %)				<0.001			<0.001
No	252,779 (75.41%)	139,117 (74.56%)	113,662 (76.47%)		146,575 (81.13%)	106,204 (68.73%)	
Yes	82,427 (24.59%)	47,462 (25.44%)	34,965 (23.53%)		34,100 (18.87%)	48,327 (31.27%)	
T2DM (%)				<0.001			<0.001
No	320,618 (95.65%)	177,047 (94.89%)	143,571 (96.6%)		177,186 (98.07%)	143,432 (92.82%)	
Yes	14,588 (4.35%)	9,532 (5.11%)	5,056 (3.4%)		3,489 (1.93%)	11,099 (7.18%)	
Smoking status (*n*, %)				<0.001			<0.001
Never	137,503 (41.02%)	77,019 (41.28%)	60,484 (40.70%)		77,784 (43.05%)	59,719 (38.65%)	
Ever	196,110 (58.50%)	108,535 (58.17%)	87,575 (58.92%)		102,148 (56.54%)	93,962 (60.80%)	
Missing	1,593 (0.48%)	1,025 (0.55%)	568 (0.38%)		743 (0.41%)	850 (0.55%)	
Alcohol status (*n*, %)				<0.001			<0.001
Never	14,081 (4.20%)	9,960 (5.34%)	4,121 (2.77%)		7,285 (4.03%)	6,796 (4.40%)	
Ever	320,304 (95.55%)	175,995 (94.33%)	144,309 (97.09%)		172,981 (95.74%)	147,323 (95.34%)	
Missing	821 (0.24%)	624 (0.33%)	197 (0.13%)		409 (0.23%)	412 (0.27%)	
Antihypertensives use (%)				<0.001			<0.001
No	277,333 (82.74%)	153,520 (82.28%)	123,813 (83.30%)		157,721 (87.30%)	119,612 (77.40%)	
Yes	57,495 (17.15%)	32,767 (17.56%)	24,728 (16.64%)		22,775 (12.61%)	34,720 (22.47%)	
Missing	378 (0.11%)	292 (0.16%)	86 (0.06%)		179 (0.10%)	199 (0.13%)	
Lowering lipids use (%)				<0.001			<0.001
No	291,124 (86.85%)	161,903 (86.77%)	129,221 (86.94%)		164,196 (90.88%)	126,928 (82.14%)	
Yes	43,704 (13.04%)	24,384 (13.07%)	19,320 (13.00%)		16,300 (9.02%)	27,404 (17.73%)	
Missing	378 (0.11%)	292 (0.16%)	86 (0.06%)		179 (0.10%)	199 (0.13%)	
Insulin use (%)				<0.001			<0.001
No	331,895 (99.01%)	184,416 (98.84%)	147,479 (99.23%)		179,588 (99.40%)	152,307 (98.56%)	
Yes	2,933 (0.87%)	1,871 (1.00%)	1,062 (0.71%)		908 (0.50%)	2,025 (1.31%)	
Missing	378 (0.11%)	292 (0.16%)	86 (0.06%)		179 (0.10%)	199 (0.13%)	
Educational levels (*n*, %)				<0.001			<0.001
None	51,694 (15.42%)	27,629 (14.81%)	24,065 (16.19%)		23,834 (13.19%)	27,860 (18.03%)	
High school and below	150,473 (44.89%)	80,949 (43.39%)	69,524 (46.78%)		80,126 (44.35%)	70,347 (45.52%)	
College or above	112,595 (33.59%)	66,723 (35.76%)	45,872 (30.86%)		66,280 (36.68%)	46,315 (29.97%)	
Other professional qualifications	16,667 (4.97%)	8,965 (4.80%)	7,702 (5.18%)		8,602 (4.76%)	8,065 (5.22%)	
Unknown or missing	3,777 (1.13%)	2,313 (1.24%)	1,464 (0.99%)		1,833 (1.01%)	1,944 (1.26%)	
Average total household income, £ (*n*, %)				<0.001			<0.001
<30,999	208,548 (62.21%)	117,331 (62.89%)	91,217 (61.37%)		108,613 (60.12%)	99,935 (64.67%)	
≥30,999	79,922 (23.84%)	43,889 (23.52%)	36,033 (24.24%)		47,482 (26.28%)	32,440 (20.99%)	
Missing	46,736 (13.94%)	25,359 (13.59%)	21,377 (14.38%)		24,580 (13.60%)	22,156 (14.34%)	
Frailty status				<0.001			<0.001
Non-frailty	192,278 (57.36%)	100,773 (54.01%)	91,505 (61.57%)		108,160 (59.86%)	84,118 (54.43%)	
Pre-frailty	114,473 (34.15%)	66,846 (35.83%)	47,627 (32.04%)		58,714 (32.5%)	55,759 (36.08%)	
Frailty	8,318 (2.48%)	6,046 (3.24%)	2,272 (1.53%)		3,423 (1.89%)	4,895 (3.17%)	
Unknown	20,137 (6.01%)	12,914 (6.92%)	7,223 (4.86%)		10,378 (5.74%)	9,759 (6.32%)	
Season of blood collection				<0.001			<0.001
Spring	98,929 (29.51%)	67,602 (36.23%)	31,327 (21.08%)		53,583 (29.66%)	45,346 (29.34%)	
Summer	88,087 (26.28%)	33,522 (17.97%)	54,565 (36.71%)		47,762 (26.44%)	40,325 (26.1%)	
Autumn	79,861 (23.82%)	36,632 (19.63%)	43,229 (29.09%)		43,223 (23.92%)	36,638 (23.71%)	
Winter	68,329 (20.38%)	48,823 (26.17%)	19,506 (13.12%)		36,107 (19.98%)	32,222 (20.85%)	

TDI, townsend deprivation index; BMI, body mass index; DBP, diastolic blood pressure; SBP, systolic blood pressure; MET-min/week, metabolic equivalent of task-minutes per week; TC, total cholesterol; HbA1c, hemoglobin A1c; HDL-C, high-density lipoprotein cholesterol; LDL-C, low-density lipoprotein cholesterol; T2DM, type 2 diabetes mellitus.

### Association of TyG and vitamin D status with MACE risk

**Figure 1:**
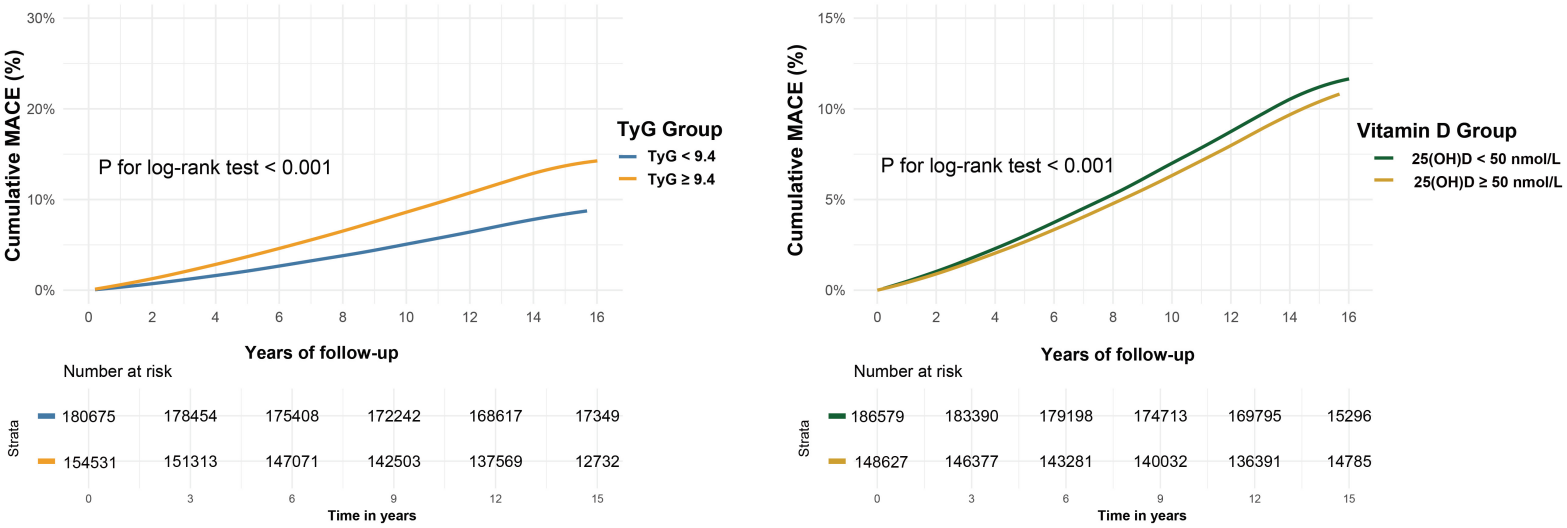
*Kaplan-Meier curves for cumulative incidence of MACE stratified by TyG index and vitamin D status*. Kaplan-Meier curves depict the cumulative incidence of MACE during follow-up, stratified by **(A)** TyG index and **(B)** serum 25(OH)D concentration. Participants with an elevated TyG index (≥9.4) and vitamin D deficiency (<50 nmol/L) exhibited a significantly higher cumulative MACE risk. Log-rank tests revealed statistically significant differences between strata (*P* < 0.001). The number-at-risk is provided below each panel. MACE, major adverse cardiovascular events.

Over a median follow-up of 13.77 years (IQR: 12.93–14.50), 33,532 participants developed MACE, including 25,692 cases of IHD, 7,917 strokes, 1,600 cardiac arrests, and 4,217 cardiovascular deaths. Kaplan–Meier analysis revealed a higher cumulative incidence of MACE in participants with TyG ≥ 9.4 compared to those with TyG <9.4, and in those with 25(OH)D <50 nmol/L compared to those with 25(OH)D ≥ 50 nmol/L (all P for log-rank test <0.001; Figure 1). The highest event rate was observed in participants with both elevated TyG index and low vitamin D status.

In unadjusted Cox models, TyG ≥ 9.4 was associated with a 70% increased risk of MACE (HR: 1.70, 95% CI: 1.66–1.73), while 25(OH)D ≥ 50 nmol/L was associated with a modestly lower risk (HR: 0.92, 95% CI: 0.90–0.94). After adjustment for demographic, clinical, and lifestyle covariates, the association between high TyG and MACE remained significant (HR: 1.17, 95% CI: 1.14–1.20), and the inverse association with higher vitamin D levels also persisted (HR: 0.90, 95% CI: 0.88–0.92), as shown in Table 2. When TyG and vitamin D were treated as continuous variables, a 1-unit increase in TyG index was associated with a 19% higher risk of MACE (adjusted HR: 1.19, 95% CI: 1.17–1.22), while each 10-nmol/L increase in 25(OH)D was associated with a 3.1% lower risk (HR: 0.969, 95% CI: 0.964–0.975). Likewise, per standard deviation increase, TyG was associated with a 10% increased risk (HR: 1.10, 95% CI: 1.09–1.12), and vitamin D was associated with a 6% lower risk of MACE (HR: 0.94, 95% CI: 0.93–0.95) in the fully adjusted model.

**Table 2:** Multivariate cox regression analysis of the association between changes in TyG and 25(OH)D levels and the risk of MACE.

TyG index	Unadjusted model	Model 1	Model 2	Model 3
Per unit increase	1.73 (1.70–1.76)^***^	1.45 (1.42–1.47)^***^	1.21 (1.19–1.24)^***^	1.19 (1.17–1.22)^***^
Per SD increase	1.36 (1.35–1.37)^***^	1.23 (1.22–1.24)^***^	1.12 (1.10–1.13)^***^	1.10 (1.09–1.12)^***^
TyG <9.4	Reference	Reference	Reference	Reference
TyG ≥ 9.4	1.70 (1.66–1.73)^***^	1.38 (1.35–1.41)^***^	1.19 (1.16–1.22)^***^	1.17 (1.14–1.20)^***^
25(OH)D (nmol/L)				
Per 10-unit increase	0.973 (0.968–0.978)^***^	0.947 (0.942–0.952)^***^	0.970 (0.964–0.975)^***^	0.969 (0.964–0.975)^***^
Per SD increase	0.94 (0.93–0.95)^***^	0.89 (0.88–0.90)^***^	0.94 (0.93–0.95)^***^	0.94 (0.93–0.95)^***^
25(OH)D <50	Reference	Reference	Reference	Reference
25(OH)D ≥ 50	0.92 (0.90–0.94)^***^	0.83 (0.81–0.85)^***^	0.90 (0.88–0.92)^***^	0.90 (0.88–0.92)^***^

^***^P < 0.001.

Model 1: Adjusted for sex, race, and age. Model 2: Adjusted for sex, race, age, BMI, MET, HbA1c, and diet score. Model 3: Adjusted for hypertension, diabetes mellitus, lipid-lowering medication, insulin use, smoking status, alcohol status, sex, race, age, BMI, MET, HbA1c, and diet score.

RCS analysis revealed significant non-linear associations between the TyG index and serum 25(OH)D levels with MACE risk. The association between the TyG index and MACE was positive and nonlinear (*P* for non-linearity = 0.012, Supplementary Figure S3), with the hazard of MACE increasing as TyG levels increased and tending to plateau at higher TyG levels. In contrast, the association between serum 25(OH)D and MACE exhibited an L-shaped pattern (*P* for non-linearity <0.001), with a steep decline in hazard across the lower range of vitamin D, followed by a flattening of the curve at higher concentrations.

### Joint association of the TyG index and vitamin D status with incident MACE risk

The joint association between the TyG index and serum vitamin D levels with MACE risk was evaluated using the group with TyG <9.4 and 25(OH)D ≥ 50 nmol/L as the reference. A graded increase in MACE risk was observed across the remaining three exposure groups (*P* for trend <0.001; Table 3).

**Table 3:** Joint association of TyG index and 25(OH)D concentration with the risk of MACE.

TyG index	Unadjusted model		Multivariate regression	
TyG <9.4 and 25(OH)D ≥ 50 nmol/L	Reference		Reference	
TyG <9.4 and 25(OH)D <50 nmol/L	0.99 (0.95–1.02)	0.413	1.06 (1.03–1.10)^***^	<0.001
TyG ≥ 9.4 and 25(OH)D ≥ 50 nmol/L	1.61 (1.56–1.67)	<0.001	1.13 (1.09–1.17)^***^	<0.001
TyG ≥ 9.4 and 25(OH)D <50 nmol/L	1.73 (1.68–1.78)	<0.001	1.26 (1.18–1.35)^***^	<0.001
*P* for trend		<0.001		<0.001

^***^P < 0.001.

HbA1c, hemoglobin A1c; BMI, body mass index.

Multivariate regression model adjusted for hypertension, diabetes mellitus, lipid-lowering medication, insulin use, smoking status, alcohol status, sex, race, age, BMI, physical activity, HbA1c, and diet score.

**Figure 2:**
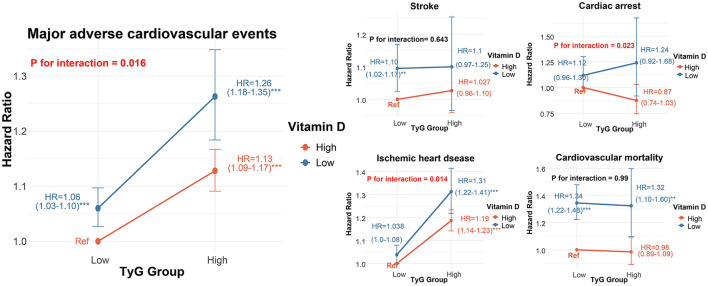
*Association of the TyG index with MACE and its components, stratified by vitamin D status*. Associations between TyG index and MACE, as well as its components, stratified by serum vitamin D status. Adjusted hazard ratios (HRs) and 95% confidence intervals (CIs) are shown for combinations of low/high TyG and low/high vitamin D. Interaction *P*-values assess whether the TyG index effect varies by vitamin D level. MACE, major adverse cardiovascular events.

In the fully adjusted Cox regression model, both low vitamin D and high TyG levels were independently associated with elevated MACE risk (Figure 2). Compared to the reference group, the HR was 1.06 (95% CI: 1.03–1.10) for participants with TyG <9.4 and 25(OH)D <50 nmol/L, and 1.13 (95% CI: 1.09–1.17) for those with TyG ≥ 9.4 and 25(OH)D ≥ 50 nmol/L. The highest risk was observed in participants with both high TyG and low vitamin D levels (HR: 1.26, 95% CI: 1.18–1.35).

### Interactions of the TyG index and vitamin D status with MACE Risk

The effects of the TyG index and vitamin D levels on MACE risk were significant in both additive and multiplicative models. In the additive model, a notable effect was observed (RERI: 0.074, 95% CI: 0.017–0.131, *P* = 0.01; AP: 0.059, 95% CI: 0.014–0.104, *P* = 0.011; SI: 1.394, 95% CI: 1.006–1.782, *P* = 0.019; Figure 2 and Supplementary Table S1), indicating that high TyG and low vitamin D status together substantially heightened MACE risk. This effect was further validated by a significant multiplicative interaction in the multiplicative model (OR: 1.056, 95% CI: 1.01–1.103, *P* = 0.016), confirming the combined influence of TyG and vitamin D on MACE risk.

Significant interactions were also identified for individual MACE components, particularly for IHD in both models (additive: RERI: 0.088, multiplicative OR: 1.065, 95% CI: 1.013–1.121, *P* = 0.014), suggesting a marked joint effect. A significant multiplicative interaction was observed for cardiac arrest (OR: 1.269, 95% CI: 1.033–1.557, *P* = 0.023). However, no significant interaction was detected for stroke (*P* = 0.643) or cardiovascular mortality (*P* = 0.99).

### Public health implications of the joint effects of TyG and vitamin D on MACE risk

Generally, individuals with high TyG were at increased MACE risk compared to those with low TyG, irrespective of vitamin D status (Table 4). Specifically, the high TyG and high vitamin D group exhibited a significantly higher risk of MACE (IRR: 1.29, 95% CI: 1.25–1.33), with 1,631.6 attributable cases and an EIR of 20.8 per 10,000 PY. In contrast, the high TyG and low vitamin D group demonstrated an even higher risk (IRR: 1.55, 95% CI: 1.50–1.60), with 4,369.9 attributable cases and a significantly increased EIR of 35.4 per 10,000 PY. The low TyG and low vitamin D group presented a modest risk (IRR: 1.12, 95% CI: 1.08–1.16), with 753 attributable cases and a lower EIR of 6.1 per 10,000 PY.

**Table 4:** Joint association of TyG index and vitamin D levels with MACE and its components: multivariate analysis of incidence rates, attributable cases, and screening requirements.

Events	Group^*^	Observed no. of events	Person-year (PY)	IRR (95% CI)^†^	Attributable cases^†^	EIR (95% CI) per 10,000 PY^†^	Number needed to screen per PY^†^
MACE	TyG high and VitD high	7,305	784,220.2	1.29 (1.25–1.33)	1,631.6 (1,440.5–1,816.5)	20.8 (18.4–23.2)	480.6 (431.7–544.4)
	TyG low and VitD low	7,032	1,231,746.1	1.12 (1.08–1.16)	753.0 (540.2–958.8)	6.1 (4.4–7.8)	1,635.8 (1,284.7–2,280)
	TyG high and VitD low	12,312	1,232,726.5	1.55 (1.50–1.60)	4,369.9 (4,130.3–4,602.4)	35.4 (33.5–37.3)	282.1 (267.8–298.5)
Cardiac arrest	TyG high and VitD high	286	828,478.4	1.02 (0.87–1.19)	NA	NA	NA
	TyG low and VitD low	358	1,273,494	1.2 (1.03–1.39)	58.7 (10.2–100.4)	0.5(0. −0.1)	21,691.7 (12,680.1–124,528.7)
	TyG high and VitD low	627	1,307,906.4	1.6 (1.40–1.83)	235.7 (179.5–284.9)	1.8 (1.4–2.2)	5,548.2 (4,590.1–7,288.1)
Stroke	TyG high and VitD high	1,675	820,534.2	1.12 (1.04–1.19)	175.3 (71.5–272.3)	2.1 (0.9–3.3)	4,681.8 (3,013.6–11,469.7)
	TyG low and VitD low	1,838	1,264,930.3	1.13 (1.06–1.20)	209.0 (99.4–311.6)	1.7 (0.8–2.5)	6,053.4 (4,059.1–12,727.4)
	TyG high and VitD low	2,592	1,296,720.6	1.25 (1.18–1.33)	526.1 (397.4–647.3)	4.1 (3.1–5.0)	2,464.8 (2,003.3–3,263.3)
IHD	TyG high and VitD high	5,754	793,370	1.37 (1.32–1.42)	1,546.4 (1,383.5–1,703.2)	19.5 (17.4–21.5)	513.1 (465.8–573.5)
	TyG low and VitD low	5,130	1,243,008.5	1.1 (1.06–1.15)	476.4 (291.8–654.0)	3.8 (2.3–5.3)	2,609.3 (1,900.7–4,260.5)
	TyG high and VitD low	9,734	1,248,019.7	1.64 (1.58–1.69)	3,787.1 (3,580.2–3,986.9)	30.3 (28.7–31.9)	329.5 (313.0–348.6)
CVM	TyG high and VitD high	820	826,068.6	1.19 (1.08–1.32)	133.0 (61.8–197.4)	1.6 (0.7–2.4)	6,213.2 (4,184.2–13,365.7)
	TyG low and VitD low	976	1,270,666	1.45 (1.32–1.59)	302.4 (235.6–363.1)	2.4 (1.9–2.9)	4,202.5 (3,499.6–5,393.3)
	TyG high and VitD low	1,645	1,302,995.8	1.79 (1.64–1.95)	723.7 (641.2–799.5)	5.6 (4.9–6.1)	1,800.4 (1,629.8–2,032.0)

^*^TyG low and Vitamin D high group as the reference group. MACE, major adverse cardiovascular diseases. CA, cardiac arrest; CVM, cardiovascular mortality; IHD, ischemic heart diseases.

CVM, cardiovascular mortality.

IRRs, incidence rate ratios; EIRs, excess incidence rates; PY, person-years.

^†^IRRs, attributable cases EIRs, and number needed to screen per PY calculated after stratification for age and sex.

Attributable cases, excess incidence rate, and number needed to screen were not calculated when the 95% CI of the IRR included 1.00, and are therefore reported as NA. In these cases, the excess risk was not statistically significant, making the derived absolute estimates unstable and not clinically interpretable.

Similar patterns were observed in the individual MACE components, with risk consistently higher for individuals with high TyG, irrespective of vitamin D status. For cardiac arrest, the high TyG and low vitamin D group exhibited an IRR of 1.60 (95% CI: 1.40–1.83), corresponding to an EIR of 1.8 per 10,000 PY and 235.7 attributable cases. For stroke, the high TyG and low vitamin D group showed an IRR of 1.25 (95% CI: 1.18–1.33) and an EIR of 4.1 per 10,000 PY, with 526.1 attributable cases. In the same exposure category, ischemic heart disease risk was significantly elevated (IRR: 1.64, 95% CI: 1.58–1.69), with an EIR of 30.3 per 10,000 PY and 3,787.1 attributable cases. Cardiovascular mortality was highest in the high TyG and low vitamin D group (IRR: 1.79, 95% CI: 1.64–1.95), with an EIR of 5.6 per 10,000 PY and 723.7 attributable cases.

### Subgroup analyses

Subgroup analyses revealed that the interactions of TyG and vitamin D on MACE risk, as assessed by both additive and multiplicative models, were most pronounced in White individuals (RERI: 0.079, *P* = 0.012), those with BMI <30 kg/m^2^ (RERI: 0.089, *P* = 0.031), and individuals without diabetes (RERI: 0.072, *P* = 0.026). Additionally, the interactions were stronger in individuals with high physical activity (RERI: 0.081, *P* = 0.018) and low dietary risk (RERI: 0.09, *P* = 0.006), as shown in Supplementary Table S2 and Supplementary Figure S4. No significant interactions were found in non-White individuals, those with diabetes, or those with high dietary risk.

### Sensitivity analyses

The sensitivity analyses corroborated the results of the primary analysis. These analyses encompassed re-evaluation utilizing comprehensive baseline data, exclusion of participants who experienced MACE within the initial 2 years of follow-up, multiple imputation with five generated datasets, and further adjustments for variables such as season, fasting duration, eGFR, and frailty level. Across these analyses, an elevated TyG index, particularly when coupled with low vitamin D levels, consistently demonstrated a significant association with an increased risk of MACE (Supplementary Tables S3–S6).

## Discussion

Based on 13.77 years of extensive follow-up data, this study is the first to identify an interaction between the TyG index and vitamin D status in relation to cardiovascular risk. Specifically, a high TyG index (≥9.4) combined with vitamin D deficiency (<50 nmol/L) significantly increased the risk of MACE by 26%. This dual-exposure group exhibited a higher EIR and a notable rise in attributable cases, underscoring the amplified cardiovascular risk linked to combined metabolic and nutritional dysregulation.

The TyG index, recognized as a marker of insulin resistance, has been established as an independent predictor of MACE in multiple studies. Multivariate Cox regression analysis has demonstrated a significant positive association between the TyG index and MACE risk. Notably, this association remains significant and independent of traditional cardiovascular risk factors in populations with diabetes, acute coronary syndrome (ACS) and hemodialysis patients with IHD (31–33). This study supports the independent effect of the TyG index on MACE risk within the general population, further reinforcing findings from previous population-based research.

While the TyG index is traditionally calculated using fasting triglyceride and glucose measurements, the samples from the UK Biobank were collected without mandatory fasting. The utility of the non-fasting TyG index is supported by its correlations with insulin resistance markers and its predictive value for cardiovascular events in large population-based studies, including relevant analyses from the UK Biobank (34–36). Theoretically, postprandial triglyceride fluctuations could introduce random variability, resulting in non-differential misclassification and attenuation of associations, or lead to systematic overestimation if individuals with metabolic dysfunction exhibit pronounced postprandial responses. Importantly, we addressed this issue in sensitivity analyses by adjusting for the time elapsed since the last meal, and the results remained materially unchanged, indicating that postprandial variation is unlikely to have significantly biased our effect estimates. It is important to note that the TyG cut-off of 9.4 was empirically derived from this non-fasting cohort; hence, its direct comparability with fasting-based thresholds reported in the literature should be interpreted with caution. Taken together, these analyses support the robustness of the non-fasting TyG index in the current study and lend credibility to the observed interaction between the TyG index and vitamin D status in relation to MACE risk.

In recent years, numerous observational studies have established a correlation between vitamin D deficiency and an elevated risk of cardiovascular diseases, including coronary heart disease, stroke, and myocardial infarction (37–39). This study is the first to identify a significant interaction between the TyG index and vitamin D status on MACE risk. Among the 335,206 individuals analyzed, the combined exposure to a high TyG index and vitamin D deficiency was found to significantly elevate the EIR and substantially increase attributable cases. This finding indicates that the simultaneous presence of metabolic dysregulation (high TyG) and vitamin D deficiency delineates a high-risk phenotype with potential to refine cardiovascular risk estimation. Future studies with formal risk prediction models are needed to evaluate the clinical utility of integrating TyG and vitamin D into risk assessment.

In interpreting these observational findings, it is important to consider that large-scale randomized controlled trials of vitamin D supplementation, such as the Vitamin D and Omega-3 Trial (VITAL) and the Vitamin D Assessment (ViDA) study, in conjunction with comprehensive meta-analyses, have not shown a significant reduction in cardiovascular disease in general populations (40–42). These neutral outcomes imply that the observed correlation between vitamin D deficiency and MACE may primarily indicate vitamin D status as a marker of residual confounding factors, metabolic disorders or an epiphenomenon, rather than a direct causal factor (10, 25). Consequently, the current evidence does not substantiate a direct causal relationship between vitamin D intervention and reduced cardiovascular risk, leaving the clinical utility of vitamin D in cardiovascular disease prevention to be further elucidated. Nevertheless, meta-analyses of randomized trials have shown that vitamin D supplementation can improve intermediate cardiometabolic markers, including reductions in C-reactive protein (CRP), glucose markers, and triglycerides (43, 44). Importantly, these intermediate markers are integral to the biological pathways through which insulin resistance and vitamin D deficiency may collectively influence cardiovascular risk, providing a mechanistic context for the observed interaction.

Insulin resistance, as captured by the TyG index—combining triglyceride and glucose concentrations—drives endothelial damage *via* multiple pro-inflammatory pathways. This condition promotes arteriosclerosis and increases cardiovascular risk by inducing endothelial dysfunction, reducing NO synthesis, and enhancing arterial inflammation (45). Furthermore, insulin resistance facilitates leukocyte adhesion to endothelial cells *via* activation of C-X-C chemokine receptor type 4, thus amplifying inflammatory responses (46). Hyperinsulinemia associated with insulin resistance also directly impairs microvascular endothelial function by inducing oxidative stress through the NADPH oxidase system (47). Together, these pro-inflammatory and oxidative stress mechanisms compromise endothelial integrity and accelerate arteriosclerosis progression (48). In parallel, vitamin D deficiency undermines its anti-inflammatory properties, leading to increased levels of pro-inflammatory cytokines such as TNF-α, IL-6, and CRP (49). This deficiency exacerbates the chronic low-grade inflammation of the vascular wall, impairs endothelial function, and may play a role in the development of inflammatory diseases. Additionally, it promotes atherosclerotic plaque formation by upregulating the NF-κB inflammatory pathway (50). Vitamin D deficiency is also linked to the activation of the renin-angiotensin system, further aggravating atherosclerosis and endothelial dysfunction (10). Moreover, it exacerbates insulin resistance by inhibiting insulin receptor phosphorylation and reducing glucose transporter activity (51, 52). This metabolic-inflammatory cascade ultimately leads to abnormal proliferation of vascular smooth muscle cells, vascular calcification, and increased arterial stiffness, contributing to cardiovascular outcomes such as atherosclerotic plaque formation and myocardial ischemia (53). The convergence of these pathways provides a plausible framework for understanding how combined high TyG and vitamin D deficiency might amplify cardiovascular risk beyond the sum of their individual effects.

This study has several notable limitations. First, while multiple confounders have been adjusted for, residual confounding factors—such as gut microbiota composition and polymorphisms in vitamin D-binding protein—may still influence causal inferences. Second, this study utilized single baseline measurements of the TyG index and serum 25(OH)D. Although the UK Biobank is well suited for prospective analyses of long-term health outcomes, repeated measurements of these biomarkers are not routinely available for the entire cohort, restricting assessments of within-person changes over time. The reliance on a single-time-point assessment may have introduced exposure misclassification and constrained our capacity to evaluate the dynamic relationship between metabolic dysfunction, vitamin D status, and the risk of MACE. Future studies incorporating repeated biomarker measurements and time-dependent models are warranted. Third, the UK Biobank is not fully representative of the general population and is prone to healthy volunteer selection bias. Consequently, prevalence estimates and absolute risk estimates may not be directly generalizable to the broader population, particularly when considering population-level screening strategies. Therefore, our findings should primarily be interpreted as association-based evidence and require validation in more representative cohorts.

## Conclusions

In conclusion, an elevated TyG index and vitamin D deficiency are independently associated with an increased risk of MACE. Moreover, a significant statistical interaction between these two factors further exacerbates the risk of MACE in the general population. The findings suggest that the concomitance of insulin resistance and vitamin D deficiency delineates a subgroup with elevated cardiovascular risk, thereby warranting further investigation to assess its significance in risk evaluation.

## Data Availability

The original contributions presented in the study are included in the article/Supplementary material, further inquiries can be directed to the corresponding authors.
