# Supplementary material for: Insulin resistance, vitamin D status, and cardiovascular risk in a general population cohort
Source: Front Nutr. 2026 Jul 17;13:1868349. doi: 10.3389/fnut.2026.1868349 (PMC13426274; doi:10.3389/fnut.2026.1868349)
Supplement: Supplementary file 1 [file Data_Sheet_1.docx]

**Supplement**

**Table S1.** Interaction Analysis of TyG Levels and 25(OH)D Concentration with the Risk of MACE and Its Specific Components.

**Table S2.** Interaction Analysis of TyG Levels and Vitamin D Concentration with the Risk of MACE and Its Specific Components Based on Different Stratifications.

**Table S3.** Sensitivity Analysis Among Participants with Complete Baseline Data

**Table S4.** Sensitivity analysis excluding participants with MACE events within the first 2 years of follow-up

**Table S5.** Multiple imputation was performed to generate five datasets, which were subsequently pooled according to Rubin’s rules.

**Table S6**. Association between joint TyG index and 25(OH)D categories and MACE risk after additional covariate adjustment

**Figure S1.** Directed acyclic graph of the association between TyG/Vitamin D and the incidence of MACE.

**Figure S2.** Study Design and Participant Selection Flowchart.

**Figure S3.** Dose-Response Associations Between TyG Index and Serum 25(OH)D Levels with MACE Risk.

**Figure S2**. Subgroup analysis of the association between TyG index and vitamin D deficiency with MACE risk.

| **Table S1. Interaction Analysis of TyG Levels and 25(OH)D Concentration with the Risk of MACE and Its Specific Components.** | | | | | | | |
| --- | --- | --- | --- | --- | --- | --- | --- |
|  | **Additive interactive** | | | | | **Multiplicative interactive**  **OR (95%CI)** | **P value** |
|  | **Measure** | **Estimate** | **Lower** | **Upper** | **P value** |  |  |
| **MACE** | RERI | 0.074 | 0.017 | 0.131 | 0.01 | 1.056  (1.01,1.103) | 0.016 |
|  | AP | 0.059 | 0.014 | 0.104 | 0.011 |  |  |
|  | S | 1.394 | 1.006 | 1.782 | 0.019 |  |  |
| **Components** |  |  |  |  |  |  |  |
| **Cardiac arrest*** | RERI | 0.248 | -0.015 | 0.511 | 0.065 | 1.269  (1.033,1.557) | 0.023 |
|  | AP | 0.2 | -0.014 | 0.413 | 0.067 |  |  |
|  | S | -30.72 | 841.03 | -902.47 | - |  |  |
| **Stroke** | RERI | -0.021 | -0.135 | 0.093 | 0.718 | 0.979  (0.895,1.071) | 0.643 |
|  | AP | -0.019 | -0.123 | 0.085 | 0.718 |  |  |
|  | S | 0.827 | -0.005 | 1.658 | 0.711 |  |  |
| **Ischemic Heart Disease** | RERI | 0.088 | 0.022 | 0.0153 | 0.001 | 1.065  (1.013,1.121) | 0.014 |
|  | AP | 0.067 | 0.017 | 0.117 | 0.001 |  |  |
|  | S | 1.39 | 1.014 | 1.765 | 0.017 |  |  |
| **Cardiovascular mortality** | RERI | -0.006 | -0.171 | 0.159 | 0.942 | 0.999  (0.881,1.134) | 0.99 |
|  | AP | -0.005 | -0.129 | 0.12 | 0.942 |  |  |
|  | S | 0.981 | 0.489 | 1.474 | 0.941 |  |  |

*The P value was not computed since log(S) is undefined when S ≤ 0.

Additive interactive model adjusted for hypertension, diabetes mellitus, lipid-lowering medication, insulin use, smoking status, alcohol status, sex, race, age, BMI, physical activity, HbA1c, and diet score.

Multiplicative interactive model adjusted for hypertension, diabetes mellitus, lipid-lowering medication, insulin use, smoking status, alcohol status, sex, race, age, BMI, physical activity, HbA1c, and diet score, as well as the product interaction between TyG and VitD groups.

| **Table S2. Interaction Analysis of TyG Levels and Vitamin D Concentration with the Risk of MACE and Its Specific Components Based on Different Stratifications.** | | | | | | | | | | | | | | |
| --- | --- | --- | --- | --- | --- | --- | --- | --- | --- | --- | --- | --- | --- | --- |
|  | Additive interactive | | | | Multiplicative interactive  OR (95%CI) | P-value |  | Additive interactive | | | | Multiplicative interactive  OR (95%CI) | P-value |  |
|  | Measure | Estimate | Lower | Upper |  |  |  | Measure | Estimate | Lower | Upper |  |  |  |
| **Race** |  |  |  |  |  |  | **DM** |  |  |  |  | 1.027  (0.864,1.222) | 0.759 |  |
| White | RERI | 0.079 | 0.02 | 0.137 | 1.06  (1.013,1.109) | 0.012 | YES | RERI | 0.038 | -0.207 | 0.282 |  |  |  |
|  | AP | 0.062 | 0.016 | 0.108 |  |  |  | AP | 0.031 | -0.172 | 0.234 |  |  |  |
|  | S | 1.431 | 1.012 | 1.85 |  |  |  | S | 1.228 | -0.495 | 2.951 | 1.054  (1.006,1.103) | 0.026 |  |
| non-White | RERI | -0.106 | -0.464 | 0.253 | 0.895  (0.685,1.17) | 0.418 | No | RERI | 0.072 | 0.013 | 1.131 |  |  |  |
|  | AP | -0.078 | -0.328 | 0.179 |  |  |  | AP | 0.057 | 0.01 | 0.104 |  |  |  |
|  | S | 0.798 | 0.21 | 1.385 |  |  |  | S | 1.384 | 0.982 | 1.787 |  |  |  |
| **Age** |  |  |  |  |  |  | **Hypertension** |  |  |  |  |  |  |  |
| < 60 years | RERI | 0.065 | -0.03 | 0.16 | 1.034  (0.962, 1.111) | 0.368 | Yes | RERI | 0.062 | -0.031 | 0.154 | 1.058 (0.986,1.135) | 0.118 |  |
|  | AP | 0.046 | -0.021 | 0.112 |  |  |  | AP | 0.055 | -0.027 | 0.138 |  |  |  |
|  | S | 1.182 | 0.882 | 1.481 |  |  |  | S | 2.112 | -0.971 | 5.196 |  |  |  |
| ≥ 60 years | RERI | 0.025 | -0.047 | 0.097 | 1.018 (0.962,1.077) | 0.535 | No | RERI | 0.084 | 0.012 | 0.157 | 1.056  (0.998,1.118) | 0.06 |  |
|  | AP | 0.021 | -0.041 | 0.097 |  |  |  | AP | 0.063 | 0.009 | 0.117 |  |  |  |
|  | S | 1.187 | 0.574 | 1.8 |  |  |  | S | 1.33 | 0.979 | 1.681 |  |  |  |
| **Sex** |  |  |  |  |  |  | **Physical activity** |  |  |  |  |  |  |  |
| Men | RERI | 0.029 | -0.043 | 0.101 | 1.022 (0.965,1.081) | 0.458 | High PA | RERI | 0.081 | 0.017 | 0.145 | 1.062  (1.01,1.117) | 0.018 |  |
|  | AP | 0.025 | -0.036 | 0.087 |  |  |  | AP | 0.064 | 0.014 | 0.114 |  |  |  |
|  | S | 1.215 | 0.608 | 1.821 |  |  |  | S | 1.434 | 0.985 | 1.883 |  |  |  |
| Women | RERI | 0.062 | -0.012 | 0.135 | 1.047 (0.989,1.109) | 0.111 | Low PA | RERI | 0.064 | -0.063 | 0.19 | 1.049  (0.954,1.154) | 0.323 |  |
|  | AP | 0.051 | -0.01 | 0.111 |  |  |  | AP | 0.052 | -0.051 | 0.155 |  |  |  |
|  | S | 1.384 | 0.796 | 1.973 |  |  |  | S | 1.394 | 0.384 | 2.404 |  |  |  |
| **BMI (Kg/m^2^)** |  |  |  |  |  |  | **Diet score** |  |  |  |  |  |  |  |
| ＜30 | RERI | 0.089 | 0.024 | 0.153 | 1.059  (1.005,1.116) | 0.031 | Low dietary risk | RERI | 0.09 | 0.027 | 0.153 | 1.071  (1.02,1.125) | 0.006 |  |
|  | AP | 0.066 | 0.018 | 0.114 |  |  |  | AP | 0.071 | 0.021 | 0.121 |  |  |  |
|  | S | 1.352 | 1.03 | 1.673 |  |  |  | S | 1.52 | 1.015 | 2.024 |  |  |  |
| ≥30 | RERI | 0.124 | 0.002 | 0.246 | 1.121  (1.026,1.226) | 0.012 | High dietary risk | RERI | 0.005 | -0.129 | 0.14 | 0.987  (0.892, 1.092) | 0.803 |  |
|  | AP | 0.108 | 0.002 | 0.214 |  |  |  | AP | 0.004 | -0.098 | 0.107 |  |  |  |
|  | S | 6.272 | -21.38 | 33.93 |  |  |  | S | 1.018 | 0.57 | 1.466 |  |  |  |

RERI, relative excess risk due to interaction; AP, attributable proportion; SI, synergy index.

Additive interactive model adjusted for hypertension, diabetes mellitus, lipid-lowering medication, insulin use, smoking status, alcohol status, sex, race, age, BMI, physical activity, HbA1c, and diet score, as well as the product interaction between TyG and VitD groups.

Multiplicative interactive model adjusted for hypertension, diabetes mellitus, lipid-lowering medication, insulin use, smoking status, alcohol status, sex, race, age, BMI, physical activity, HbA1c, and diet score, as well as the product interaction between TyG and VitD groups.

| **Table S3. Sensitivity Analysis Among Participants with Complete Baseline Data** | | |
| --- | --- | --- |
| TyG index | Multivariate regression P-value | |
| TyG < 9.4 and 25(OH)D ≥ 50 nmol/L | Reference |  |
| TyG < 9.4 and 25(OH)D < 50 nmol/L | 1.05 (1.01-1.10)^***^ | 0.013 |
| TyG ≥ 9.4 and 25(OH)D ≥ 50 nmol/L | 1.11 (1.06-1.15)^***^ | < 0.001 |
| TyG ≥ 9.4 and 25(OH)D < 50 nmol/L | 1.26 (1.21-1.30)^***^ | < 0.001 |
| P for trend | < 0.001 | |

Multivariate regression has djusted for hypertension, diabetes mellitus, lipid-lowering medication, insulin use, smoking status, alcohol status, sex, race, age, BMI, MET, HbA1c, and diet score.

| **Table S4. Sensitivity analysis excluding participants with MACE events within the first 2 years of follow-up** | | |
| --- | --- | --- |
| TyG index | Multivariate regression P-value | |
| TyG < 9.4 and 25(OH)D ≥ 50 nmol/L | Reference |  |
| TyG < 9.4 and 25(OH)D < 50 nmol/L | 1.05 (1.01-1.09)^***^ | 0.006 |
| TyG ≥ 9.4 and 25(OH)D ≥ 50 nmol/L | 1.15 (1.08-1.16)^***^ | < 0.001 |
| TyG ≥ 9.4 and 25(OH)D < 50 nmol/L | 1.26 (1.22-1.30)^***^ | < 0.001 |
| P for trend | < 0.001 | |

Multivariate regression has djusted for hypertension, diabetes mellitus, lipid-lowering medication, insulin use, smoking status, alcohol status, sex, race, age, BMI, MET, HbA1c, and diet score.

| **Table S5. Multiple imputation was performed to generate five datasets, which were subsequently pooled according to Rubin’s rules.** | | |
| --- | --- | --- |
| TyG index | Multivariate regression P-value | |
| TyG < 9.4 and 25(OH)D ≥ 50 nmol/L | Reference |  |
| TyG < 9.4 and 25(OH)D < 50 nmol/L | 1.06 (1.02-1.09)^***^ | 0.001 |
| TyG ≥ 9.4 and 25(OH)D ≥ 50 nmol/L | 1.13 (1.09-1.17)^***^ | < 0.001 |
| TyG ≥ 9.4 and 25(OH)D < 50 nmol/L | 1.26 (1.22-1.30)^***^ | < 0.001 |
| P for trend | < 0.001 | |

Multivariate regression has djusted for hypertension, diabetes mellitus, lipid-lowering medication, insulin use, smoking status, alcohol status, sex, race, age, BMI, MET, HbA1c, and diet score.

| **Table S6. Association between joint TyG index and 25(OH)D categories and MACE risk after additional covariate adjustment** | | | | | | | | |
| --- | --- | --- | --- | --- | --- | --- | --- | --- |
| TyG index | **Multivariate regression ^a^** | | **Multivariate regression ^b^** | | **Multivariate regression ^c^** | | **Multivariate regression ^d^** | |
|  | HR | P-value | HR | P-value | HR | P-value | HR | P-value |
| TyG < 9.4 and 25(OH)D ≥ 50 nmol/L | Reference |  |  |  |  |  |  |  |
| TyG < 9.4 and 25(OH)D < 50 nmol/L | 1.07 (1.04-1.11)^***^ | < 0.001 | 1.07 (1.03-1.11)^***^ | < 0.001 | 1.07 (1.03-1.11)^***^ | < 0.001 | 1.06 (1.02-1.09) | 0.002 |
| TyG ≥ 9.4 and 25(OH)D ≥ 50 nmol/L | 1.13 (1.09-1.17)^***^ | < 0.001 | 1.13 (1.10-1.17)^***^ | < 0.001 | 1.13 (1.10-1.17)^***^ | < 0.001 | 1.14 (1.10-1.18)^***^ | < 0.001 |
| TyG ≥ 9.4 and 25(OH)D < 50 nmol/L | 1.28 (1.24-1.32)^***^ | < 0.001 | 1.28 (1.24-1.33)^***^ | < 0.001 | 1.28 (1.24-1.33)^***^ | < 0.001 | 1.27 (1.23-1.31)^***^ | < 0.001 |
| P for trend | < 0.001 | | < 0.001 | | < 0.001 | | < 0.001 | |

^a^ Multivariable regression was adjusted for hypertension, diabetes mellitus, lipid-lowering medication, insulin use, smoking status, alcohol status, sex, race, age, BMI, MET, HbA1c, diet score, and season.

^b^ Multivariable regression was additionally adjusted for fasting time.

^c^ Multivariable regression was additionally adjusted for eGFR.

^d^ Multivariable regression was additionally adjusted for frailty level.

**Figure S1.** Directed acyclic graph of the association between TyG/Vitamin D and the incidence of MACE.


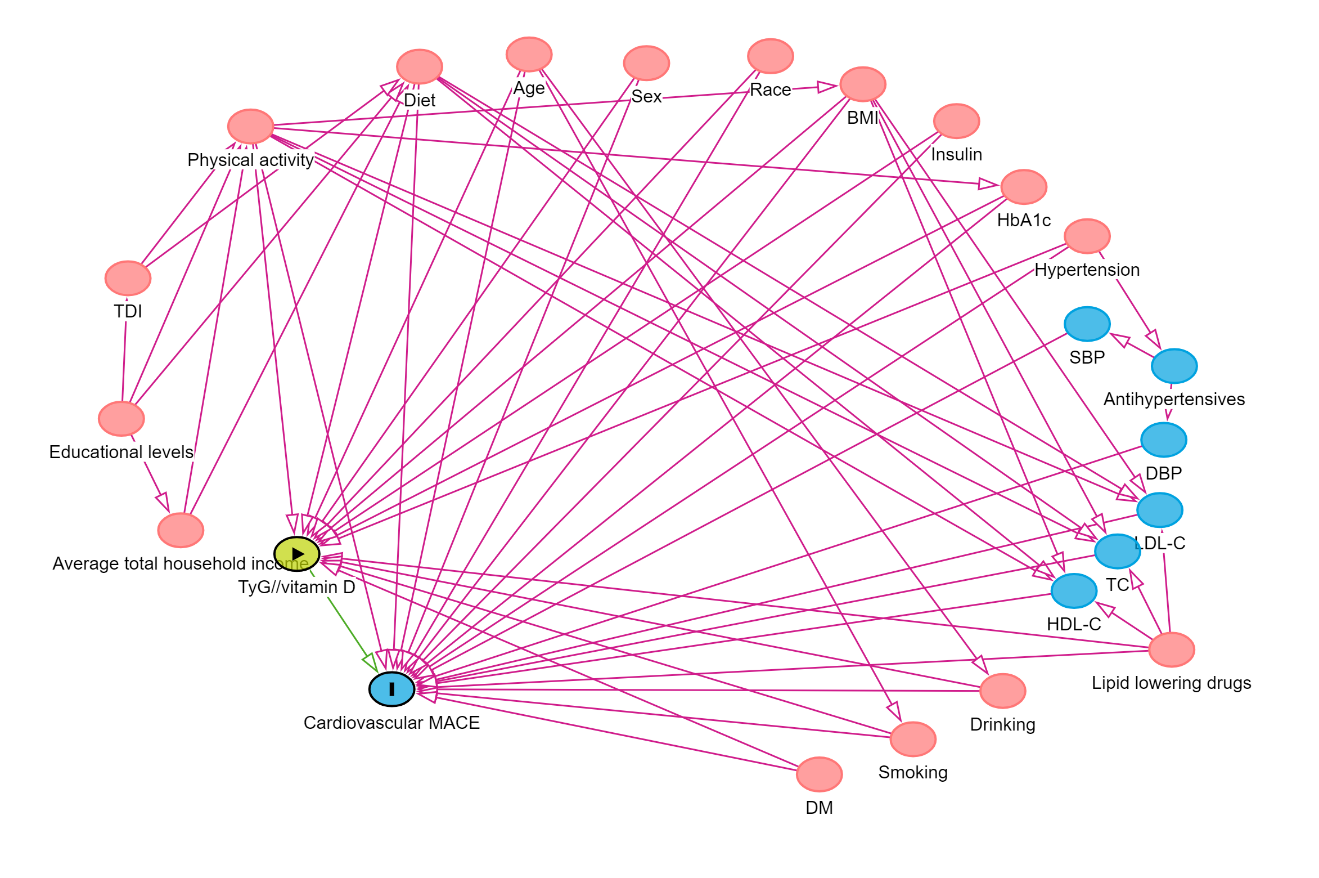


Abbreviations: BMI, body mass index; TDI, Townsend Deprivation Index; BMI : Body mass index; DBP, Diastolic Blood Pressure; SBP, Systolic blood pressure; TyG index, triglycerides-glucose index. DM, diabetes mellitus.

Notes:

The graph was created with the help of DAGitty.net (www.dagitty.net). Minimally sufficient adjustment age, sex, race, BMI, physical activity, diet score, HbA1c, hypertension, DM, insulin use, lipid-lowering medication, smoking status, and alcohol consumption

**Figure S2.** Study Design and Participant Selection Flowchart.


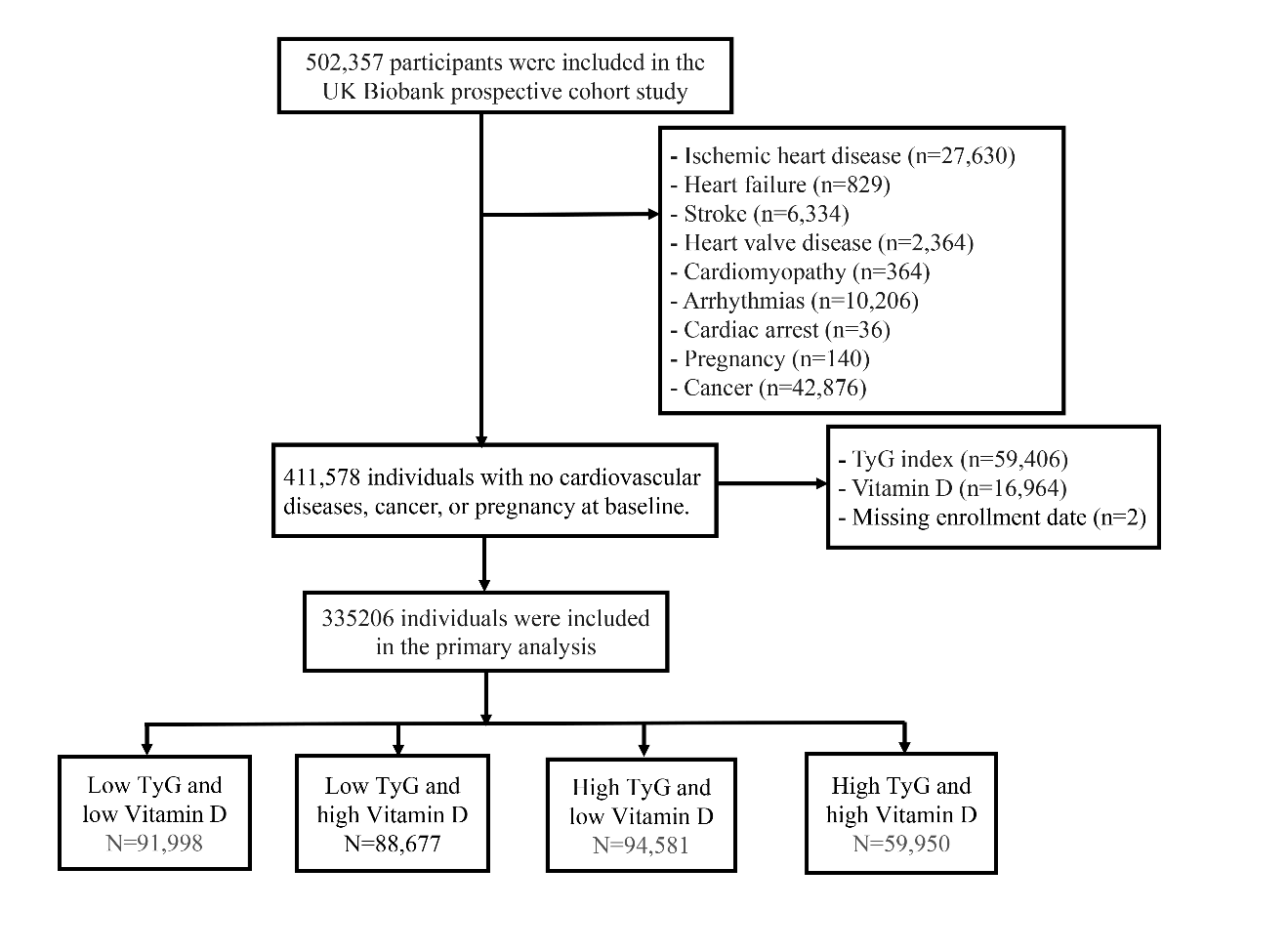


**Figure S3.** Dose-Response Associations Between TyG Index and Serum 25(OH)D Levels with MACE Risk.
Restricted cubic spline models illustrate the nonlinear relationship between (A) TyG index and (B) serum 25(OH)D levels with MACE risk. Red lines represent hazard ratios (HRs), with shaded areas indicating 95% confidence intervals (CIs). The background grey histograms show the distribution of the population (%) across TyG and 25(OH)D levels (plotted on the right y-axis). P-values reflect the significance of the nonlinear component in the spline model. MACE, Major Adverse Cardiovascular Events.


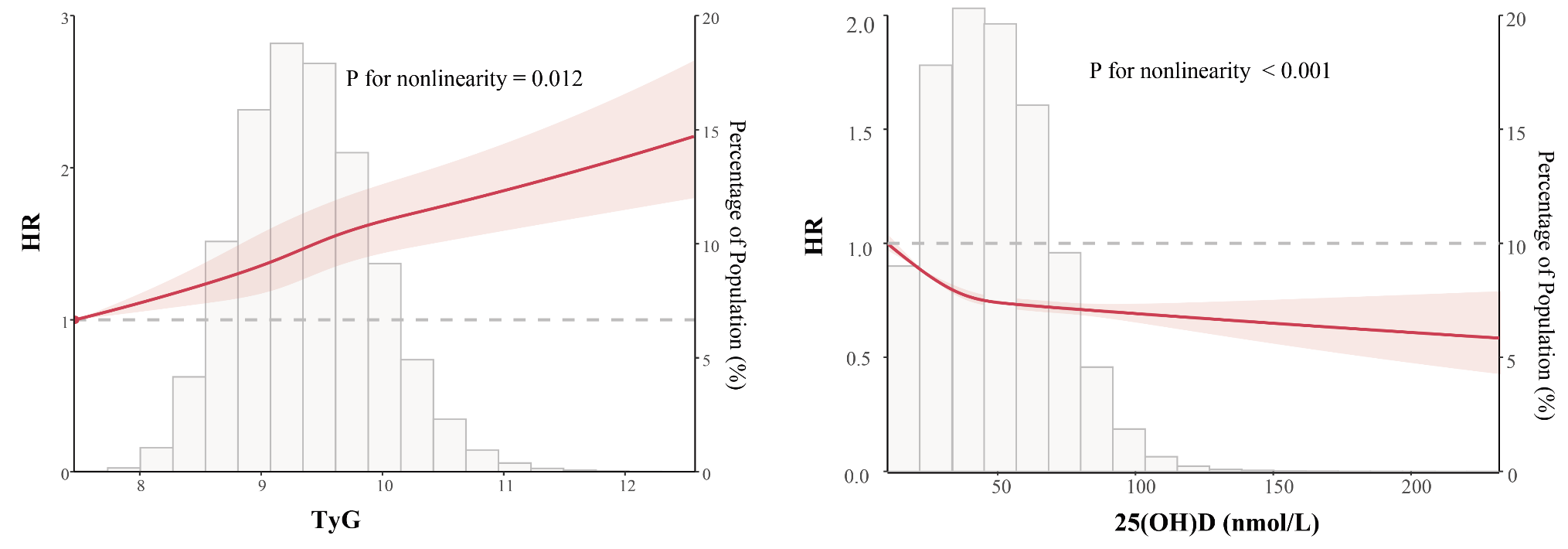


**Figure S4**. Subgroup analysis of the association between TyG index and vitamin D deficiency with MACE risk.

MACE, major adverse cardiovascular events.


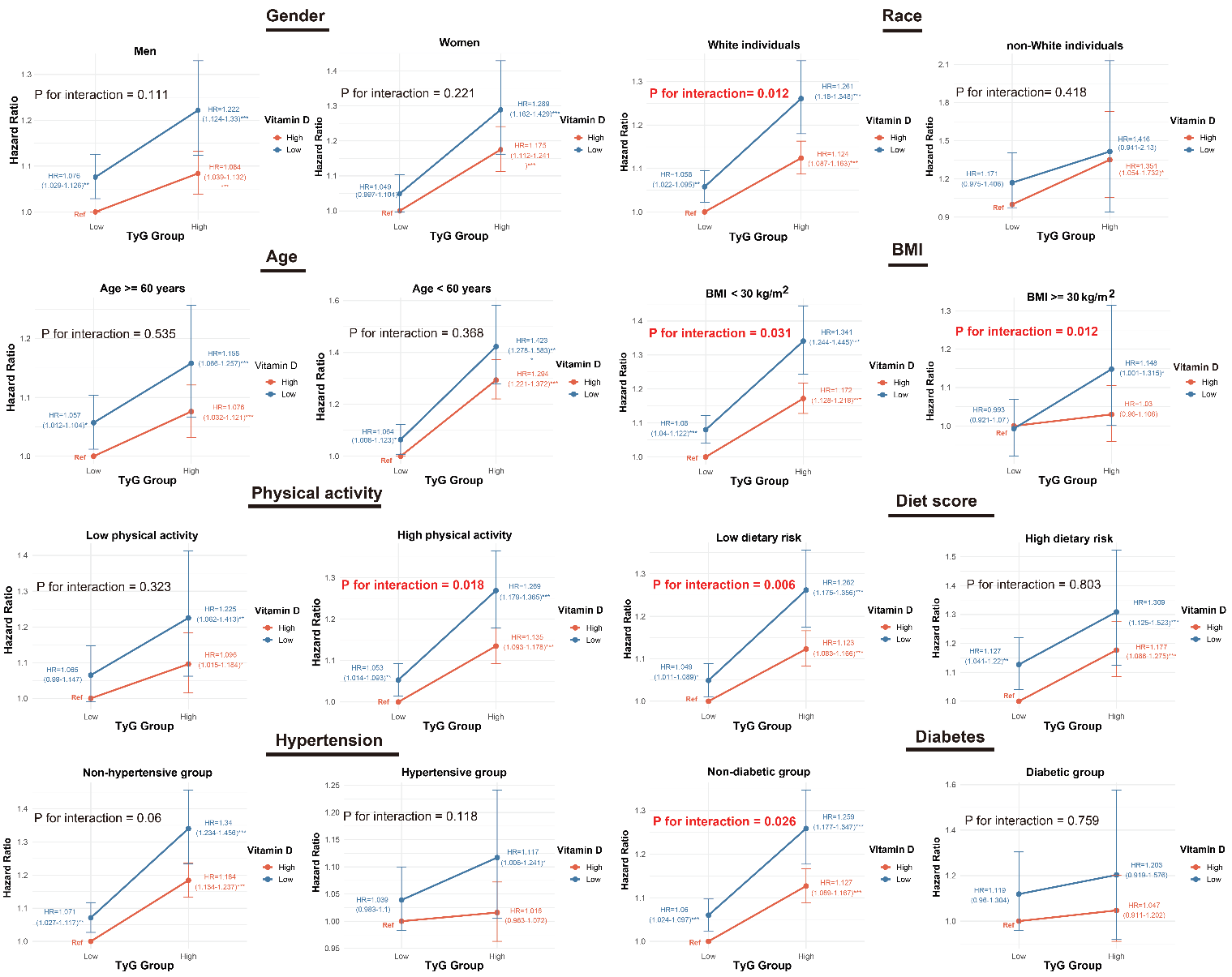


Multiplicative interactive model adjusted for hypertension, diabetes mellitus, lipid-lowering medication, insulin use, smoking status, alcohol status, sex, race, age, BMI, physical activity, HbA1c, and diet score, as well as the product interaction between TyG and VitD groups.

^*^P < 0.05; ^**^ P < 0.01; ^***^P < 0.001
